# MiR-148a-3p Regulates Skeletal Muscle Satellite Cell Differentiation and Apoptosis via the PI3K/AKT Signaling Pathway by Targeting Meox2

**DOI:** 10.3389/fgene.2020.00512

**Published:** 2020-06-04

**Authors:** Huadong Yin, Haorong He, Xinao Cao, Xiaoxu Shen, Shunshun Han, Can Cui, Jing Zhao, Yuanhang Wei, Yuqi Chen, Lu Xia, Yan Wang, Diyan Li, Qing Zhu

**Affiliations:** Farm Animal Genetic Resources Exploration and Innovation Key Laboratory of Sichuan Province, Sichuan Agricultural University, Chengdu, China

**Keywords:** miR-148a-3p, Meox2, PI3K/AKT pathway, skeletal muscle satellite cell, differentiation

## Abstract

As bioinformatic approaches have been developed, it has been demonstrated that microRNAs (miRNAs) are involved in the formation of muscles and play important roles in regulation of muscle cell proliferation and differentiation. Previously, it has been demonstrated that miR-148a-3p is one of the most abundant miRNAs in chicken skeletal muscle. Here, we build on that work and demonstrate that miR-148a-3p is important in the control of differentiation of chicken skeletal muscle satellite cells (SMSCs). Elevated expression of miR-148a-3p significantly promoted differentiation and inhibited apoptosis of SMSCs but did not affect proliferation. Furthermore, it was observed that the mesenchyme homeobox 2 (Meox2) is a target gene of miR-148a-3p and that miR-148a-3p can down-regulate expression of Meox2, which promote differentiation of SMSCs and suppress apoptosis. Furthermore, miR-148a-3p overexpression encouraged activation of the PI3K/AKT signaling pathway, which could be recovered by overexpression of Meox2. Overall, these findings suggest that microRNA-148a-3p is a potent promoter of myogenesis via direct targeting of Meox2 and increase of the PI3K/AKT signaling pathway in chicken SMSCs.

## Introduction

Skeletal muscle is the most abundant striated muscle tissue accounting for 40–60% of adult animal body weight, plays a vital role in initiating movement, maintains homeostasis, and supports respiration. Additionally, loss of skeletal muscle function can lead to aging of muscles and development of various diseases (Han et al., 2019b). Furthermore, skeletal muscle is important economically in meat-producing animals (Davoli et al., 2011). It is one of the important indicators to measure the economic value of agricultural animals. MicroRNAs (miRNAs) have been shown to be involved in muscle formation and play an important role in regulating muscle cell growth and muscle disease (Ma et al., 2015). Therefore, our results may provide a theoretical basis for RNA treatment of muscle diseases. Myogenesis is a complex and tightly regulated process, which controls proliferation, cell cycle arrest, and differentiation of somatic cells to mature muscle tissue (Han et al., 2019a). As a complex multi-step process, myogenesis is driven by a variety of signaling pathways including phosphatidylinositol-3-kinase (PI3K)/AKT, and regulatory factors including DNA, non-coding RNAs, and peptides (Guo et al., 2018).

MiRNAs are small non-coding RNAs (approximately 22 nucleotides long) that play a fundamental role in regulation of development and metabolism through posttranscriptional gene silencing. Evidence exists that suggests that up to one-third of genes might be targeted by one or more miRNAs, while comprising less than 2% of all eukaryotic genes (Krek et al., 2005). miRNAs recognize and target mRNA using a 7 nt seed sequence and reduce expression of the target proteins related genes by inhibiting mRNA translation or promoting mRNA decay (Bartel, 2009). In addition, miRNAs exert regulatory controls in some unconventional ways, such as miRNAs in the nucleus activating gene transcription and down regulating other non-coding RNAs (Dragomir et al., 2018).

The role of miR-148a in cancer cell proliferation, apoptosis, and metastasis was first demonstrated in tumor types such as cholangiocarcinoma (Lujambio et al., 2008), esophageal (Hummel et al., 2011), and colon cancers (Chen et al., 2010). Subsequent miRNA transcriptome profiling using solexa deep sequencing demonstrated that miR-148a is the most abundant miRNAs in swine and chicken skeletal muscles (Li et al., 2011; Hou et al., 2012), suggesting a potential role in skeletal muscle development. Additionally, Zhang et al. (2012) identified miR-148a as a novel myogenic miRNA that promotes myogenic differentiation by directly targeting the 3′ UTR of ROCK1 in C2C12 myoblast and primary muscle cells. Furthermore, Song et al. (2019) proposed that miR-148a-3p inhibits bovine myoblast cell proliferation and promotes apoptosis through posttranscriptional downregulation of KLF6. In previous work from our laboratory, it was demonstrated that miR-148a-3p is abundantly expressed in skeletal muscle of embryonic chickens (NCBI accession number PRJNA516545). However, molecular function and target genes of miR-148a-3p in controlling proliferation and differentiation of chicken muscle cells remains unclear. Recent bioinformatics work has highlighted the growth homology arrest specificity homology box (Gax), otherwise known as mesenchyme homeobox 2 (Meox2/Mox2) as a miRNAs of interest in the regulation of limb muscle formation in vertebrates (Mankoo et al., 1999).

In the current study, we assessed regulatory mechanisms of miR-148a-3p in the proliferation and differentiation of skeletal muscle satellite cells (SMSCs) in chickens via a series of experiments.

## Materials and Methods

### Animal and Ethics Standards

The ROSS 308 broiler chickens were purchased from the Yunda Poultry Breeding Cooperative (Xinjin County, Sichuan Province, China). For each cell collection, five to eight ROSS 308 broilers were used. A total of 10 times were collected, and about 60 broilers were used. Animal care and experimental procedures were approved by the Animal Welfare Committee of the Faculty of Agriculture at the Sichuan Agriculture University (assurance number 2018-007-01).

### Culturing of Cells

The chest muscles of 4 days old ROSS 308 chicks were collected for primary isolation and culture of SMSCs. Muscles were first cut and then treated with 0.1% collagenase I (Sigma, MO, United States) followed by 0.25% trypsin (Hyclone, UT, United States) to release cells. The cell suspension was then filtered and subjected to Percoll density centrifugation to isolate myocytes. Cells were plated in 25 cm^3^ cell culture bottles with complete medium [DMEM/F12 (Invitrogen, Carlsbad, CA, United States) + 15% FBS (Gibco, NY, United States) + 1% penicillin–streptomycin (Solarbio, Beijing, China) + 3% chicken embryo extraction], which were allowed to proliferate in growth medium for 2–4 days, and the medium was refreshed every 24 h. To induce differentiation, satellite cells are cultured in a differentiation medium consisting of DMEM/F12, 2% horse serum (Hyclone, UT, United States), and 1% penicillin–streptomycin when satellite cells grow to 80% confluence in growth medium, and the medium was refreshed every 24 h. The negative control was used as the control condition in the experiment. The chicken blast fibroblast cell line DF-1 was used for the dual luciferase reporter assay, and DF-1 was cultured in DMEM (FBS, Hyclone, United States) supplemented with 10% (v/v) fetal bovine serum (Gibco, United States). Cells were cultured at 37°C in a 5% CO_2_ humidified atmosphere, and medium was refreshed every 24 h.

### Construction of Plasmids and RNA Oligonucleotides

The miR-148a-3p inhibitor, negative inhibitor, miR-148a-3p mimic, and negative mimic were purchased from RiboBio (RiboBio, Guangzhou, China). siRNA for chicken Meox2 were purchased from GenePharma (GenePharma, Shanghai, China). Coding sequences (CDS) of Meox2 were amplified from chicken genomic DNA using polymerase chain reaction (PCR). Coding sequence fragments of Meox2 were ligated into pcDNA3.1 (+) vector using T4 DNA ligase following restrictive endonuclease digestion. Following validation, successfully constructed vectors were termed pcDNA3.1-Meox2. Detailed sequences are provided in Table 1.

**TABLE 1:** RNA oligonucleotide and plasmid construction in this article.

Name	Sequence (5′–3′)	Accession number
miR-148a-3p mimic	UCAGUGCACUACAGAACUUUGU	miRBase: MIMAT0001120
Negative mimic	UUGUACUACACAAAAGUACUG	
miR-148a-3p inhibitor	ACAAAGUUCUGUAGUGCACUGA	
Negative inhibitor	CAGUACUUUUGUGUAGUACAA	
Si-Meox2	GCAGUAAACCUUGACCUCATT UGAGGUCAAGGUUUACUGCTT	NCBI: NM_001005427.1
Si-NC	UUCUCCGAACGUGUCACGUTT ACGUGACACGUUCGGAGAATT	
pcDNA3.1-Meox2	GACACGGATCCGCCACCATGGATCA CACACTCTTTG GTGTCCTCGAGTCATAAGTGTGCGTG	

### Transfection of Cells

MiR-148a-3p mimics, inhibitors, si-Meox2, or pcDNA3.1-Meox2 were transfected into satellite cells using Lipofectamine^®^ 3000 (Invitrogen, Carlsbad, CA, United States) according to the manufacturer’s instructions, and repeated three times per group. When the cell fusion rate reached about 80–90%, transfected cells were cultured in DM to study cell differentiation; 50–60% of transfected cells were cultured in GM to study cell proliferation and apoptosis. Dilute oligonucleotides or plasmids, Lipofectamine^®^ 3000, and diluted DNA using Opti-MEM^®^ (Gibco, Langley, OK, United States) media and transfect into cells. Samples were collected after 24 h (proliferation/apoptosis) or 48 h (differentiation) for further analysis.

### Extraction of RNA, Synthesis of cDNA, and Real-Time Quantitative PCR

According to the manufacturer’s instructions, TRIzol reagent (Invitrogen) was used to extract total RNA from satellite cells for detection of expression of genes related to proliferation/differentiation/apoptosis or identification of transfection efficiency. The selection of marker genes for proliferation/differentiation/apoptosis is determined based on previous literature, and these genes all play an important role in their respective processes. As for the stability of genes, it is because of this that we have selected four genes for each process for testing. Integrity and concentration of RNA in samples were measured using the Thermo Scientific^TM^ NanoDrop Lite (Thermo Fisher Scientific, Waltham, MA, United States). Total RNA was stored at −80°C. Reverse transcription of mRNA was performed using PrimeScript RT Master Mix Perfect Real Time (Takara, Dalian, China), and reverse transcription reactions for miRNA were performed using the One Step miRNA cDNA Synthesis Kit as per the manufacturer’s instructions (HaiGene, Haerbin, China).

Real-time PCR primers were designed using Primer Premier 6 and are listed in Table 2. Abundance of mRNA for each gene was measured using CFX96-Touch^TM^ real-time PCR detection system (Bio-Rad, Hercules, CA, United States). The specific reaction volume ratio of real-time PCR is: cDNA/reverse and forward primers/double distilled water/TB Green^TM^ Premix Ex Taq^TM^ II (Takara, Dalian, China) = 2/1/6/10. All reactions were performed in triplicate. Relative gene expression was determined by the 2^–ΔΔCt^ method.

**TABLE 2:** Primers used for quantitative real-time PCR.

Gene	Primer sequences (5′–3′)	Product size (bp)	Accession number	TM (°C)
β-actin	F: GTCCACCGCAAATGCTTCTAA R: TGCGCATTTATGGGTTTTGTT	78	NM_205518.1	58
MyoG	F: CGTGTGCCACAGCCAATG R: CCGCCGGAGAGAGACCTT	63	NM_204184.1	60
MyoD1	F: GCCGCCGATGACTTCTATGA R: CAGGTCCTCGAAGAAGTGCAT	66	NM_204214.1	60
MYHC	F: GAAGGAGACCTCAACGAGATGG R: ATTCAGGTGTCCCAAGTCATCC	138	XM_015295778.2	60
Myf5	F: CCTCATGTGGGCTTGCAAA R: CCTTCCGCCGGTCCAT	59	NM_001030363.1	59
PCNA	F: AACACTCAGAGCAGAAGAC R: GCACAGGAGATGACAACA	225	NM_204170.2	55
CDK2	F: CCAGAACCTCCTCATCAAC R: CAGATGTCCACAGCAGTC	171	NM_001199857.1	55
CCND1	F: CTCCTATCAATGCCTCACA R: TCTGCTTCGTCCTCTACA	165	NM_205381.1	54
Ki67	F: GCAACAACAAGGAGGCTTCG R: TTCAGGTGCCATCCCGTAAC	204	XM_025151669.1	60
Meox2	F: GAGGAAAAGCGACAGCTCAGAT R: TCTCTGATTTGCTCCTTGGTGA	105	NM_001005427.1	60
Caspase-3	F: TGGCCCTCTTGAACTGAAAG R: TCCACTGTCTGCTTCAATACC	139	NM_204725.1	58
Caspase-9	F: GCTTGTCCATCCCAGTCCAA R: CAGTCTGTGGTCGCTCTTGT	95	XM_424580.6	60
miR-148a-3p	F: TCAGTGCACTACAGAACTTTGT R: CAGGTCCAGTTTTTTTTTTTTTT		MIMAT0001120 (miRBase)	56
U6	F: GGGCCATGCTAATCTTCTCTGTA R: CAGGTCCAGTTTTTTTTTTTTTT			56

In order to investigate the extensiveness and difference of miR-148a-3p and Meox2 profile expression in different chicken tissues, the heart, stomach, intestine, liver, brain, chest, and leg muscles of 4 days old ROSS 308 broiler chickens were collected, using the remaining samples of the time of cell collection. Tissues were ground in liquid nitrogen and then TRIzol reagent (Invitrogen) was used for RNA extraction. Include the following procedures, cDNA synthesis, and analysis.

### CCK-8 Assay

Cell proliferation was measured using Cell Count Kit-8 (Meilunbio, Shanghai, China) according to the manufacturer’s instructions, and cell suspensions (100 μl/well) were seeded in 96-well plates. Place the plate in the incubator for a period of time (37°C, 5% CO_2_), add 10 μl of CCK-8 solution to each well, and incubate the plate in the incubator for 1–4 h. Repeat six times for each group. Absorbance was measured at 450 nm using a Thermo Scientific^TM^ Varioskan LUX. Four time points were taken for measurement, which were 12, 24, 48, and 72 h after transfection.

### EdU Assay

After transfection of cells, EDU experiments were performed using the C10310 EdU Apollo *in vitro* imaging kit (RiboBio, Guangzhou, China) according to the manufacturer’s instructions. Cells were first incubated with EdU medium and then washed with PBS (phosphate-buffered saline). Next, the cells were then fixed with 4% paraformaldehyde and then the cells were stained using a kit. Three areas were randomly selected using a fluorescence microscope to assess the number of stained cells.

### Immunofluorescence

According to the manufacturer’s instructions, cells were fixed using 4% paraformaldehyde on glass coverslips and washed three times using PBS for 3 min. Fixed cells were permeabilized using 0.5% Triton X-100 for 20 min at room temperature and blocked with goat serum for 30 min. Next, cell diluted primary antibody was added and incubated overnight at 4°C. Slides were diluted using PBST (0.05% Tween 20 + PBS) three times and diluted secondary antibody was added, and incubated at 20–37°C for 1 h. Next, cell nuclei were stained using DAPI (4′,6-diamidino-2-phenylindole) in the dark for 5 min. Images were taken using a fluorescence microscope.

### Western Blot

The collected transfected cells were extracted with total protein using a total protein extraction kit (BestBio, Shanghai, China). Determine the sample concentration according to the instructions of the BCA Protein Quantification Kit (BestBio, Shanghai, China) and calculate the optimal sample load. Next, electrophoresis (SDS-PAGE) is performed. After the sample is added, electrophoresis is performed at a voltage of 60 V. When the sample is completely in the separation gel, the voltage is adjusted to 120 V. The next step is to carefully remove the electrophoresed gel for electrophoresis transfer, and set the voltage and time according to the instructions of the Bio-Rad semi-dry membrane transfer instrument. Then incubate and wash the primary and secondary antibodies. The primary antibody is incubated at 37°C for 2 h, and the secondary antibody is incubated at 37°C for 1.5 h. Finally, use the ECL method to detect, observe, and take pictures.

Antibodies used for experiments included anti-Myosin (Santa Cruz Biotechnology, CA, United States; 1:200 dilution), anti-MyoG (Santa Cruz Biotechnology; 1:500 dilution), anti-caspase-3 (Bioworld, United States; 1:1,000 dilution), anti-caspase-9 (Abcam, London, United Kingdom; 1:1,000 dilution), anti-AKT, anti-p-AKT, anti-β-actin (Santa Cruz Biotechnology; 1: 1,000 dilution), and anti-GAPDH (ZENBIO, China, 1: 5,000 dilution). β-actin and GAPDH were used as a loading control.

### Luciferase Reporter Assay

Fragments of miR-148a-3p, including the binding site of Meox2, were amplified and inserted into pEZX-FR02 vectors (GeneCopoeia, United States) at the 3′ end of the Firefly Luciferase gene using restriction enzymes *Bsi*WI and *Xho*I (TaKaRa, Dalian, China) and T4 DNA ligase (pEZX-Meox2-WT). Mutant pEZX-Meox2-MT was generated by mutating complementary to the seed region of miR-148a-3p using mutagenic primers. All constructs were verified by sequence analysis.

### Measurement of Apoptosis

A 300-mesh nylon gauze was used to filter the SMSC suspension. Following filtration, SMSC suspensions were washed using pre-chilled PBS twice. Cells were resuspended in 1 × binding buffer (BD Pharmingen, Santiago, CA, United States) to attain a final cell density of 1 × 10^6^ cells/ml. Next, 100 μl of solution was transferred to a culture tube, and 5 μl of PI (BD Pharmingen) and Annexin V-FITC was added (BD Pharmingen), respectively. After mixing, cells were incubated at 25°C for 15 min in the dark, then 400 μl of 1 × binding buffer (BD Pharmingen) was added to each tube and measured using a flow cytometer (BD Bioscience).

### Prediction of Target Gene

Target genes of miR-148a-3p were predicted using TargetScan^1^, miRDB^2^, and Diana^3^.

### Statistical Analysis

Statistical analyses were performed using SPSS 19.0 Statistical software (SPSS, Inc., Chicago, IL, United States). Each experiment was repeated three times. One-way analysis of variance or paired *t*-test was used to test statistical significance between groups. Data are presented as least squares means ± standard error of the mean (SEM). Differences were considered significant at the *P* < 0.05 level (^∗^*P* < 0.05, ^∗∗^*P* < 0.01).

## Results

### Expression of miR-148a-3p During Proliferation and Differentiation of SMSCs

**FIGURE 1:**
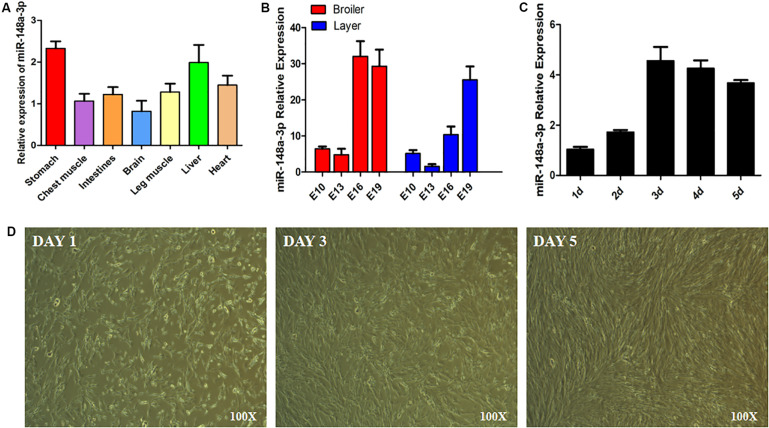
Expression of miR-148a-3p in SMSCs. **(A)** Expression of miR-148a-3p in tissues of chickens. **(B)** Expression of miR-148a-3p in chest muscle during embryo development of ROSS 308 (broiler) and White Leghorn (layer). **(C)** Expression profile over time of miR-148a-3p from 1 to 5 days of satellite cell culturing. **(D)** Morphological changes in chicken skeletal muscle satellite cells after 1, 3, and 5 days.

Expression of miR-148a-3p was observed in chest muscle, leg muscle, heart, liver, and stomach of chickens (Figure 1A). In addition, we measured greater expression of miR-148a-3p in the chest muscle of chicken embryos later in the embryonic development period (Figure 1B). Chicken SMSCs were used as a model for the identification of functional characteristics of miR-148a-3p in skeletal muscles. Results demonstrated that SMSC differentiation began on the 3rd day, while expression of miR-148a-3p was significantly up-regulated during cell differentiation (Figures 1C,D).

### Expression of miR-148-3p in SMSCs

**FIGURE 2:**
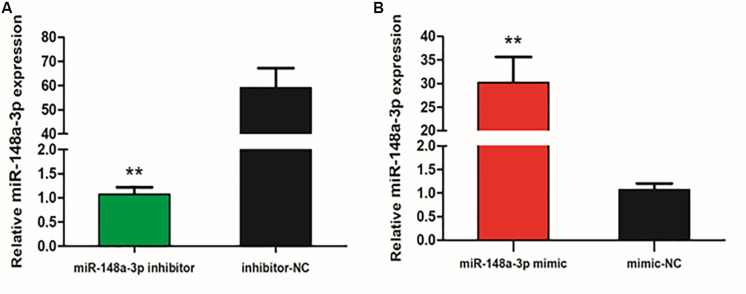
Expression of miR-148-3p as modulated by a miR-148-3p mimic or inhibitor. **(A,B)** Expression of miR-148-3p in SMSCs was monitored using qRT-PCR following transfection with a miR-148-3p inhibitor or mimic. Data are presented as mean ± SEM of three individuals. The Student’s *t*-test was used to compare expression levels among different groups. **P < 0.01 vs. NC.

To explore the function of miR-148-3p in chicken SMSCs, we modulated expression of miR-148-3p. It was observed that expression of miR-148-3p was 60 times less following transfection with the miR-148-3p inhibitor (*P* < 0.01; Figure 2A), while an increase in expression of 30-fold was observed with the miR-148-3p mimic (*P* < 0.01; Figure 2B).

### Proliferation of SMSCs Following Transfection With miR-148a-3p

**FIGURE 3:**
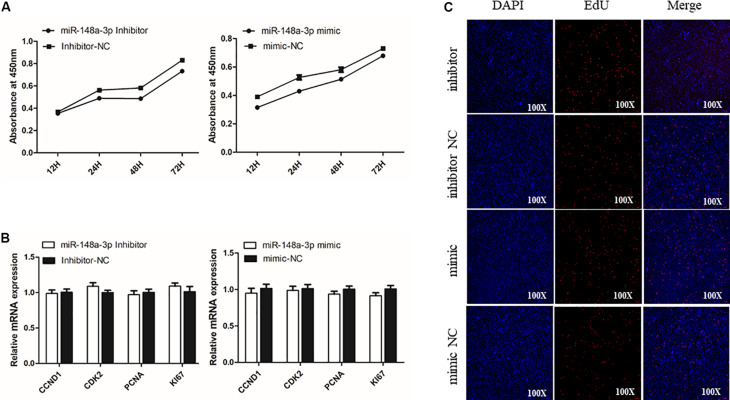
Effect of miR-148a-3p on the proliferation of SMSCs. **(A)** Cell growth curves as measured using the CCK-8 assay following transfection with a miR-148a-3p mimic, inhibitor or negative control in SMSCs. **(B)** Results of Edu assay for SMSCs transfected with a miR-148a-3p mimic, inhibitor, or negative control, where EdU (red) fluorescence is used as an indicator of proliferation and nuclei are indicated by Hoechst (blue) fluorescence. Photomicrographs were taken using a 100× magnification. **(C)** mRNA expression of CCND1 and PCNA in SMSCs transfected with a miR-148a-3p mimic, inhibitor, or negative control. Data are expressed as mean ± SEM (*N* = 3).

To assess potential roles of miR-148a-3p in SMSC proliferation, we transfected SMSCs cultured in growth medium (GM) with a miR-148-3p mimic, inhibitor, or negative control for 0, 24, 48, and 72 h. The CCk-8 assay was used to monitor transfected cells and demonstrated near linear growth (Figure 3A). Thus, results indicate that miR-148a had no significant effect on SMSC proliferation. In addition, cell proliferation was monitored using the EdU assay and demonstrated that the ratio of EdU-positive cells in the control was similar to miR-148-3p knockdown and overexpressed cells. Furthermore, mRNA expression of the cell proliferation-related gene cyclin D1 (CCND1) and proliferating cell nuclear antigen (PCNA) were examined to elucidate the effect of miR-148-3p transfection on SMSC proliferation. It was observed that CCND1 and PCNA mRNA expression was not significantly different following transfection with the inducer or inhibitor of miR-148a-3p (*P* > 0.05; Figures 3B,C).

### MiR-148a-3p Promotes Differentiation of SMSCs

**FIGURE 4:**
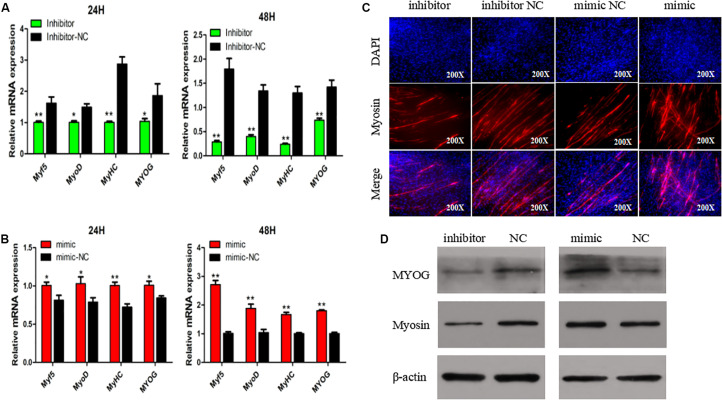
Effect of miR-148a-3p on differentiation of SMSCs. **(A,B)** Relative expression of mRNA of the genes Myf5, MyoD, MyHC, and MyoG, and abundance of proteins MyoG and Myosin as measured at 48 h of cell differentiation in SMSCs transfected with a miR-148a-3p mimic, inhibitor, or negative control. **(C)** Representative images of immunofluorescent staining of differentiated SMSCs (200×). Myosin: red, a molecular marker of myogenesis; DAPI: blue, cell nuclei; Merge: the fusion of SMSCs into primary myotubes. **(D)** Western blot detects protein levels of myogenic marker genes after inhibition and overexpression of miR-148a-3p. Data are expressed as mean ± SEM (*N* = 3). *P < 0.05; **P < 0.01 vs. NC.

To explore if SMSC differentiation is regulated by miR-148-3p, we measured mRNA expression of four gene markers of muscle differentiation. The results showed that miR-148a-3p overexpression significantly increased MyoD (24 h: 26% increase, 48 h: 88% increase), MyoG (24 h: 19% increase, 48 h: 80% increase), MyHC (24 h: increase 39%, 48 h: 67% increase), and Myf5 (24 h: 23% increase, 48 h: 171% increase) mRNA expression, while inhibition of miR-148a-3p resulted in MyoD (24 h: 33% decrease, 48 h: 71% reduction), MyoG (24 h: 46% reduction, 48 h: 51% reduction), MyHC (24 h: 65% reduction, 48 h: 82% reduction), and Myf5 (24 h: 38% reduction, 48 h: reduction 85%) reduced mRNA expression (Figures 4A,B). Furthermore, we monitored differentiation of satellite cells by use of the anti-Myosin immunofluorescence assay. Overexpression of miR-148a-3p increased differentiation of Myosin-positive cells, while inhibition of miR-148a-3p decreased differentiation of Myosin-positive cells (Figure 4C). Meanwhile, Western blot analysis demonstrated that abundance of myogenin and MyHC was greater in SMSCs transfected with the miR-148-3p mimic when compared to the negative control. Conversely, abundances of myogenin and MyHC were lesser when SMSCs were transfected with the miR-148-3p inhibitor (Figure 4D). Overall, these results suggest that miR-148a-3p promotes differentiation of SMSCs.

### MiR-148a-3p Has Negative Effect on Apoptosis of SMSC

**FIGURE 5:**
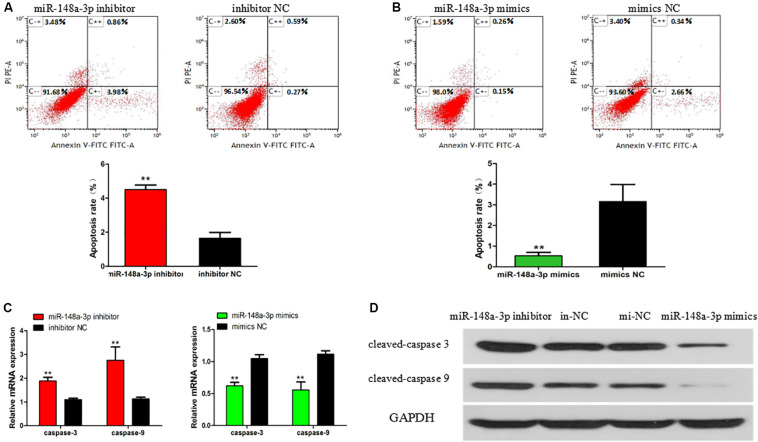
Effect of miR-148a-3p on rate of apoptosis in SMSCs. Scattergram and rate of apoptosis in SMSCs transfected with miR-148a-3p inhibitor **(A)** or miR-148a-3p mimics **(B)** as analyzed using flow cytometry following staining with annexin V and PI. **(C,D)** Abundance of mRNA and proteins of caspase 3 and caspase 9 in SMSCs transfected with miR-148a-3p inhibitor and miR-148a-3p mimics as determined by use of Western blot analysis. Data are expressed as mean ± SEM (*N* = 3). **P < 0.01 vs. NC.

The apoptosis of SMSCs is also of great significance in the development of skeletal muscle. To verify the biological effects of miR-148a-3p on the apoptosis of SMSCs, conjugated annexin V antibody and propidium iodide (PI) staining were used to analyze apoptosis by flow cytometry. After miR-148a-3p mimic transfection, the apoptosis rate of SMSC decreased by 83%, while miR-148a-3p inhibitors promoted the apoptosis of SMSC, and the apoptosis rate increased by 173% (Figures 5A,B, *P* < 0.01). In addition, we monitored the mRNA and protein abundance of the intrinsic apoptosis markers caspase 3 and caspase 9. After miR-148a-3p overexpression, caspase 3 and caspase 9 mRNA expressions decreased by 38 and 45%, and after miR-148a-3p knockdown, caspase 3 and caspase 9 mRNA expressions increased by 88 and 175%, respectively, (Figure 5C, *P* < 0.01). Western blot analysis showed that caspase 3 and caspase 9 cleavage were reduced and increased in overexpressed miR-148a-3p and silenced miR-148a-3p cells, respectively, relative to the control group (Figure 5D).

### Meox2 as a Target Gene of miR-148a-3p

**FIGURE 6:**
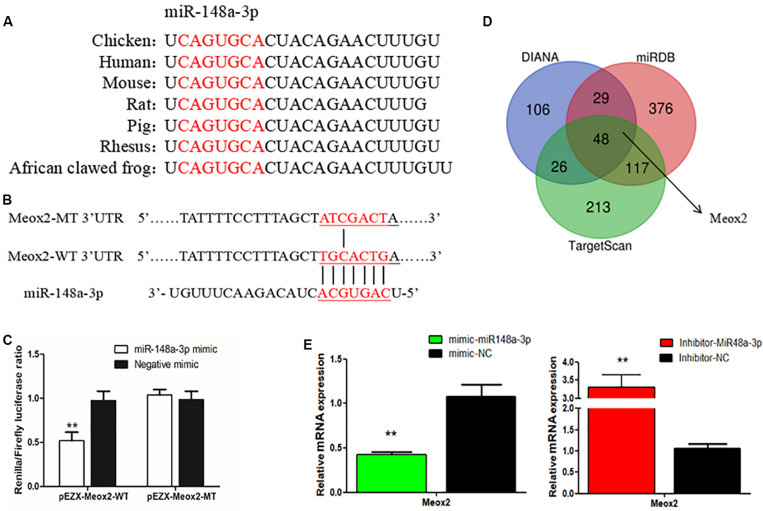
Meox2 as a target gene of miR-148a-3p. **(A)** Seed sequence of miR-148a-3p. **(B)** Prediction of target genes using TargetScan, miRDB, and Diana. **(C)** Dual-luciferase reporter gene (pEZX-FR02) with wild type (pEZX-Meox2-WT) or mutant (pEZX-Meox2-MT). **(D)** Luciferase assays were performed by co-transfection of wild-type or mutant Meox2 3′ UTR with a miR-148a-3p mimic or mimic-NC in SMSCs. **(E)** Expression of Meox2 following transfection with miR-148-30 by use of qRT-PCR. Data are expressed as mean ± SEM (*N* = 3). **P < 0.01 vs. NC.

To further elucidate mechanisms of regulation of myogenesis of SMSCs by miR-148a-3p, we identified target genes of miR-148a-3p. Using sequence alignment, we found that the seed sequence of miR-148a-3p is highly conserved (Figure 6A). Next, we used TargetScan, miRDB, and Diana to predict target genes of miR-148a-3p. Predictions made using the three online software tools were compared to identify the most likely target genes, which included Meox2 (Figure 6B). Subsequently, analysis demonstrated that that the 3′ UTR of Meox2 is a potential binding site for miR-148a-3p (Figure 6C). To verify whether miR-148a-3p has a targeting relationship with Meox2, we constructed a dual-luciferase reporter gene (pEZX-FR02) with wild type (pEZX-Meox2-WT) or mutant (pEZX-Meox2-MT) using the Meox2 3′ UTR sequence at the 3′ end of the firefly luciferase. Co-transfection of DF-1 cells with the miR-148a-3p mimic and dual-luciferase reporter gene resulted in significantly lesser activity of firefly luciferase in the pEZX-Meox2-WT plasmid. However, the miR-148a-3p mimic did not affect relative luciferase activity of the pEZX-Meox2-MT plasmid (Figure 6D). These results indicate that Meox2 is a target gene of miR-148a-3p. After transfection with miR-148a-3p mimics or inhibitors, we observed that Meox2 expression was down-regulated by 58% after miR-148a-3p overexpression, and the opposite was seen after miR-148a-3p inhibition, with a 231% increase in expression (Figure 6E).

### Meox2 Inhibits Differentiation of SMSCs

**FIGURE 7:**
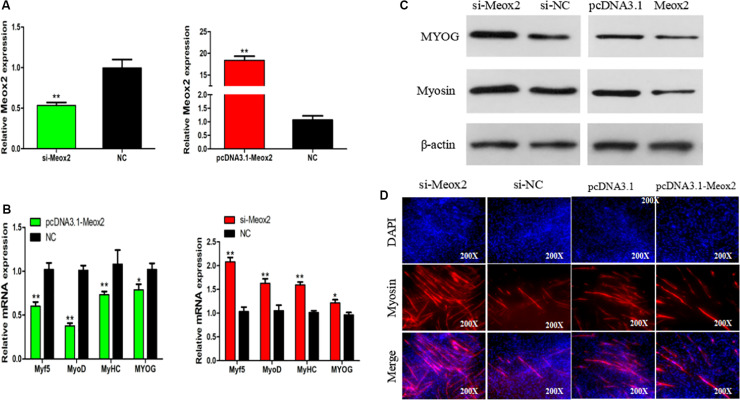
Meox2 inhibits SMSCs differentiation. **(A–C)** Expression of Meox2, MyoD, MyoG, MyHC, and Myf5 mRNA and the protein abundance of MyoG and Myosin in SMSCs transfected with si-Meox2, pCD3.1-Meox2, or negative control. **(D)** Representative images of immunofluorescent staining of differentiated SMSCs (200×). Myosin: red, a molecular marker of myogenesis; DAPI: blue, cell nuclei; Merge: the fusion of SMSCs into the primary myotubes. Data are expressed as mean ± SEM (*N* = 3). *P < 0.05; **P < 0.01 vs. NC.

In order to clarify the role of Meox2 in chicken SMSC differentiation, we synthesized a vector specific for siRNA-Meox2, and its interference and overexpression efficiency can reach 0.53 and 18 times (Figure 7A). After inhibiting Meox2, MyoD (100% increase), MyoG (63% increase), MyHC (59% increase), and Myf5 (22% increase), mRNA expression increased, while the overexpression of Meox2 in SMSC was reversed, and MyoD (40% reduction), MyoG (62% reduction), MyHC (27% reduction), and Myf5 (21% reduction) mRNA expression was reduced (Figure 7B). Western blot and immunofluorescence assay further confirmed these results and demonstrated that abundances of myogenin and MyHC increased as well as the number of myosin-positive cells following inhibition of Meox2, while the opposite was true following overexpression of Meox2 (Figures 7C,D). These results indicate that Meox2 is an inhibitor of differentiation of chicken SMSCs.

### Meox2 Promotes Apoptosis of SMSCs

**FIGURE 8:**
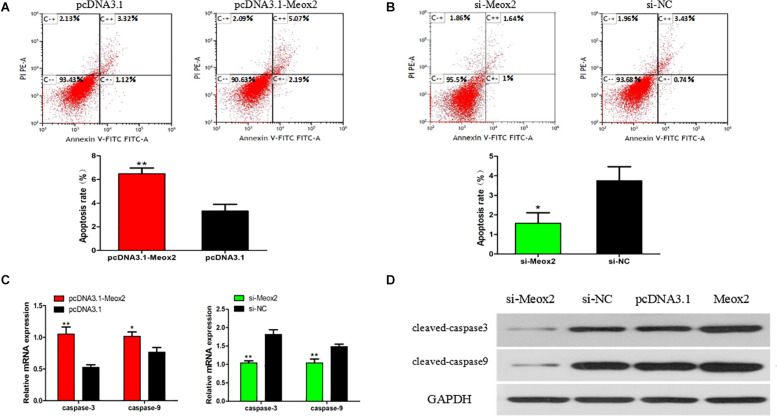
Effect of Meox2 on rate of apoptosis in SMSCs. Scattergram and rate of apoptosis in SMSCs transfected with pCD3.1-Meox2 **(A)** or siRNA-Meox2 **(B)** as analyzed using flow cytometry following staining with annexin V and PI. **(C,D)** Abundance of mRNA and proteins of caspase 3 and caspase 9 in SMSCs transfected with pCD3.1-Meox2 as determined by use of Western blot analysis. Data are expressed as mean ± SEM (*N* = 3). *P < 0.05; **P < 0.01 vs. NC.

Flow cytometry results showed that after transfecting cells with pCD3.1-Meox2, the apoptosis rate induced by SMSC increased by 95%, and the interference of Meox2 on the cells resulted in a 58% reduction in apoptosis levels (Figures 8A,B). The mRNA expression levels of caspase 3 and caspase 9 were determined by q-PCR. After Meox2 overexpression, caspase 3 and caspase 9 mRNA expression increased by 99 and 33%, and after Meox2 knockdown, caspase 3 and caspase 9 mRNA expression decreased by 45 and 32% (Figure 8C). Western blot analysis showed that the expression trend was the same as that of mRNA. Compared with the control group, overexpressed meox2 increased the expression of caspase 3 and caspase 9, while silenced Meox2 reversed (Figure 8D).

### MiR-148a-3p Affects the PI3K/AKT Signaling Pathway in SMSCs

**FIGURE 9:**
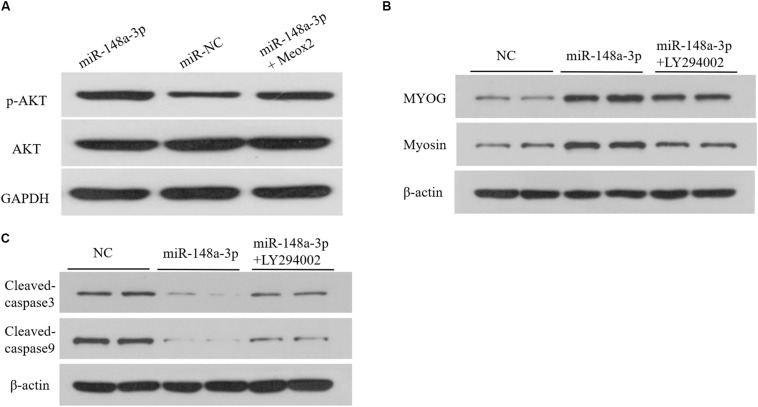
Upregulation of miR-148a-3p suppresses the PI3K/AKT signaling pathways in SMSCs. **(A)** The miR-148a-3p mimic was transfected into SMSCs with pcDNA3.1 or pcDNA3.1-Meox2. At 48 h after transfection, western blot analysis was completed to assess expression of AKT, p-AKT and GAPDH. **(B,C)** Effect of miR-148a-3p mimics on differentiation and apoptosis of SMSCs in the absence or presence of LY294002. Data are expressed as mean ± SEM (*N* = 3).

As reported previously, Meox2 is involved in regulation of the PI3K/AKT signaling pathway (Liu et al., 2015; Tian et al., 2018). To assess whether miR-148a-3p affects the PI3K/AKT signaling pathway in SMSCS, we measured expression of AKT, p-AKT in SMSCs co-transfected with a miR-148a-3p mimic, and pcDNA-3.1-Meox2 or pcDNA3.1 using Western blot analysis. Results demonstrated that ectopic expression of miR-148a-3p increased p-AKT abundances, while expression of AKT was unaltered in SMSCs. Moreover, co-transfection of pcDNA3.1-Meox2 in SMSCs restored effects of miR-148a-3p overexpression on p-AKT expression (Figure 9A). To further confirm our results, a specific PI3K/AKT inhibitor (LY294002) was used to treat cells. The results show that exogenous miR-148a-3p can increase the differentiation ability of SMSCs and inhibit apoptosis, but these effects are inhibited in SMSCs treated with pharmacological inhibitors at the same time (Figures 9B,C). These results indicate that MiR-148a-3p regulates the differentiation and apoptosis of SMSCs depending on the PI3K/AKT signaling pathway.

## Discussion

Chickens are a well-established model to study skeletal muscle formation in vertebrates, as developmental anatomy of chicken skeletal muscles shares similarities to that of mammals (Berti et al., 2015). Previously, Li et al. (2011) identified 33 novel and 189 known miRNAs involved in regulation of myogenesis by comparing miRNA transcriptomes in skeletal muscle tissues of chickens and laying hens (Li et al., 2011). Since then, a number of skeletal muscle-specific miRNAs have been identified in chicken. Lin et al. (2012) reported that let-7b is involved in regulation of the growth hormone receptor (GHR) gene, which controls skeletal muscle growth (Lin et al., 2012). In addition, let-7b has been observed to inhibit proliferation of chicken myoblasts by targeting IGF2BP3 (Lin et al., 2017). Transcriptome-wide analysis of mRNA–miRNA interactions revealed that miR-142-5p, which targets FOXO3, might contribute to regulation of skeletal muscle development in chicken (Li et al., 2018). Furthermore, the same team discovered that miR-34b-5p can mediate proliferation and differentiation of chicken myoblasts by targeting IGFBP2 (Wang et al., 2019).

Gga-miR-148a-3p (miRBase accession number: MIMAT0001120) is a mature expression form of miR-148a. To date, sequences of miR-148a-3p have been obtained in 21 species and are annotated in the miRBase (22.0) database, including *Homo sapiens*, *Mus musculus*, *Rattus norvegicus*, and *Gallus gallus* (Kozomara and Griffiths-Jones, 2013). miR-148a-3p is known for the role it plays in control of inflammation, tumors, and cancer (Takahashi et al., 2012; Yuan et al., 2012). Additionally, it has been demonstrated that miR-148a-3p is involved in the control of proliferation and differentiation of adipocytes (Tian et al., 2017; He et al., 2018). In the current study, miR-148a was of interest because it is primarily expressed in skeletal muscle of swine and chicken and regulates proliferation and differentiation of bovine and mouse myoblasts. However, to date, the role of miR-148a-3p in chicken skeletal muscle remained unclear. In the current study, the miR-148a-3p in chicken skeletal muscle differentiation was assessed by monitoring expression of mRNA and protein of the myogenic regulators Myf5, MyoD, MyoG, MyHC, and Myosion. Furthermore, immunofluorescence of myosin in SMSCs was used to confirm our result that miR-148a-3p can promote cell differentiation. However, the role of miR-148a-3p-dependent effects on cell proliferation was not clear when cell differentiation was assessed by use of the EdU or CCK-8 assays. These findings were consistent with results of Zhang et al. (2012) in which miR-148a-mediated acceleration of myogenesis was not accompanied by inhibition of cell proliferation in C2C12 myoblast cells. To the authors’ knowledge, this is the first investigation of expression and role of miR-148a-3p in chicken SMSCs.

Identification of target genes is important for functional characterization of miRNAs. It is well known that miRNAs regulate mRNA expression of target genes by binding to the 3′ untranslated region (3′ UTR), interacting with the 5′ untranslated region and impacting translation (5′ UTR) or binding to the coding region of target genes (Lytle et al., 2007; Shukla et al., 2011). Molecular targets of miR-148a-3p have been identified in several cancer cells, including muscle cells. In the present study, we identified 48 target genes of miR-148a-3p using analysis software available from TargetScan, miRDB, and Diana. The mature sequence of the miR-148a-3p matched that of the 3′ UTR of Meox2. Subsequently, results of dual luciferase assay confirmed Meox2 as a target of miR-148a-3p as overexpression and inhibition of miR-148a-3p resulted in greater and lesser expression of Meox2, respectively. These results were novel as Meox2 had not previously been identified as a molecular target of miR-148a-3p. Therefore, results of this study suggest that miR-148a-3p regulates skeletal muscle development by targeting Meox2.

After we identified 48 common goals through three bioinformatics programs, we enriched these 48 genes using DAVID online software^4^. Then search for GO functions related to muscle development or signaling pathways to determine if subsequent validation is valuable. The results show that genes worthy of verification include Meox2, PTEN, KLF4, FXR1, MDFIC, TFDP2, PPP1CB, SIK1, and VTI1A. Among them, Meox2 is enriched with GO: 0007519 (skeletal muscle tissue development) function. Interestingly, Meox2 is expressed in the somite of developing chicken embryos, in ventral and dorsal portions of limb buds, and is associated with non-proliferating myoblasts (Rallis et al., 2001). A detailed analysis of adult muscle development in the absence of Meox2 observed that Meox2 controls muscle size and muscle fiber metabolism (Otto et al., 2010). Furthermore, Meox2 contributes to myogenesis by regulating Myf5 and Pax3, which are target genes of miR-148a-3p. So, we chose Meox2 for the next step of verification. In the current study, we demonstrated a significant increase in the expression of marker genes of differentiation following inhibition of Meox2, whereas the opposite was observed following overexpression of Meox2. Conversely, the expression of the differentiation marker gene after overexpression of Meox2 was significantly reduced. Further experimental results of Western blot and immunofluorescence also showed the same trend. These results suggest that miR-148a-3p promotes the differentiation of SMSCs by inhibiting expression of Meox2.

PI3K/AKT is a critical cell signaling pathway involved in a number of cellular functions including survival, differentiation, growth, protein expression, and skeletal muscle myogenesis (Briata et al., 2012; Xi et al., 2015). Previously, it has been demonstrated that miRNAs abundant in muscles can regulate myoblast differentiation using the PI3K/AKT signaling pathway. For example, Bai et al. (2015) demonstrated that the novel miRNA miRNA-21 is involved in skeletal muscle development and regulates PI3K/Akt/mTOR signaling by targeting the TGFβI gene. Furthermore, Ma et al. (2017) identified microRNA-432 as a potent inhibitor of myogenesis through its targeting of E2F3 and P55PIK via the PI3K/AKT/mTOR signaling pathway. Huang et al. (2019) demonstrated that miR-146b-3p regulates proliferation, differentiation, and apoptosis of myoblast via direct suppression of the PI3K/AKT pathway in chickens. Because the PI3K/Akt pathway and Meox2 contribute to the regulation of myoblast differentiation, miR-148a-3p might target Meox2 to exert its inhibitory effects. In the current study, Western blot analysis demonstrated that levels of phosphorylation of AKT are activated when transfected with the miR-148a-3p mimic and that these effects had a declining effect when Meox2 was overexpressed. These results suggest that miR-148a-3p inhibits activation of the PI3K/AKT signaling pathway by regulating Meox2 in SMSCs.

**FIGURE 10:**
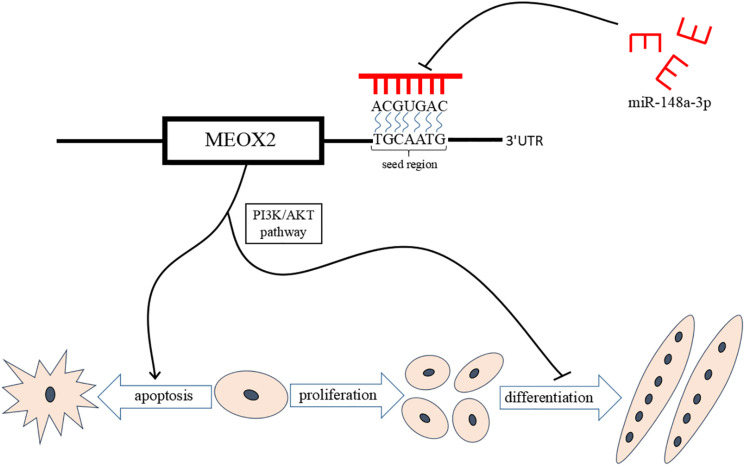
miR-148a-3p-mediated skeletal muscle satellite cell regulatory pathway model. miR-148a-3p activates the PI3K/AKT pathway by directly inhibiting Meox2 expression, and then miR-148a-3p promotes differentiation of SMSCs and inhibits apoptosis of SMSCs by inhibiting Meox2.

Results of our study provide further evidence of the mechanism by which miR-148a-3p promotes differentiation of chicken SMSCs and inhibits SMSCs apoptosis through promoting PI3K/AKT signaling pathway activity and Meox2 expression. These results support a novel model for regulation of skeletal muscle development by miR-148a-3p. A better understanding of the role of miR-148a-3p in myogenesis provides opportunities for the better control of meat quality in the animal production industry and human medical research (Figure 10).

## Data Availability Statement

The datasets generated for this study can be found in the NCBI accession number PRJNA516545.

## Ethics Statement

The animal study was reviewed and approved by the Animal Welfare Committee of the Faculty of Agriculture at the Sichuan Agriculture University. Written informed consent was obtained from the owners for the participation of their animals in this study.

## Author Contributions

XS and YWe: conceptualization. HH: data curation. HY, XC, SH, and YWe: formal analysis. HY and QZ: funding acquisition. HH, XC, and JZ: investigation. HH and XS: methodology. SH and CC: project administration. YC and LX: resources. XS: software. LX, JZ, and YWa: supervision. CC, YC, and DL: validation. YWa and DL: visualization. HY and HH: writing – original draft. QZ: writing – review and editing.

## Conflict of Interest

The authors declare that the research was conducted in the absence of any commercial or financial relationships that could be construed as a potential conflict of interest.

## Footnotes

1 http://www.targetscan.org/vert_71/

2 http://mirdb.org/

3 http://www.microrna.gr/microT-CDS

4 https://david.ncifcrf.gov/
